# Human Intestinal Parasite Burden and Poor Sanitation in Rural Alabama

**DOI:** 10.4269/ajtmh.17-0396

**Published:** 2017-09-05

**Authors:** Megan L. McKenna, Shannon McAtee, Patricia E. Bryan, Rebecca Jeun, Tabitha Ward, Jacob Kraus, Maria E. Bottazzi, Peter J. Hotez, Catherine C. Flowers, Rojelio Mejia

**Affiliations:** 1 Departments of Pediatrics and Medicine, National School of Tropical Medicine, Baylor College of Medicine, Houston, Texas;; 2 Alabama Center for Rural Enterprise, Montgomery, Alabama

## Abstract

Hookworm infection affects 430 million people worldwide, causing iron deficiency, impaired cognitive development, and stunting in children. Because of the environmental conditions needed for the hookworm life-cycle, this parasite is endemic to resource-limited countries. *Necator americanus* was endemic in the southern United States before improvement of sewage disposal systems and eradication programs. With continued poverty, poor sanitation, and an environment suitable for the hookworm life-cycle in some regions of the southern United States, a current prevalence study using modern molecular diagnostics is warranted. Lowndes County, Alabama, was chosen as the study site given previous high hookworm burdens, degree of poverty, and use of open-sewage systems. Participants were interviewed, and stool, serum, and soil samples were tested for nine intestinal parasites using a multiparallel quantitative real-time PCR (qPCR) and enzyme-linked immunosorbent assays. We found that, among 24 households, 42.4% reported exposure to raw sewage within their home, and from 55 stool samples, 19 (34.5%) tested positive for *N. americanus*, four (7.3%) for *Strongyloides stercoralis*, and one (1.8%) for *Entamoeba histolytica*. Stool tested positive for *N. americanus* contained low levels of parasite DNA (geometric mean 0.0302 fg/μL). Soil studies detected one (2.9%) *Cryptosporidium* species, and *Toxocara* serology assay detected one (5.2%) positive in this population. Individuals living in this high-risk environment within the United States continue to have stool samples positive for *N. americanus*. Gastrointestinal parasites known to be endemic to developing countries are identifiable in American poverty regions, and areas with lower disease burden are more likely to be identified by using qPCR.

## INTRODUCTION

Intestinal parasitic infections have a significant impact on health outcomes and morbidity in adults and children worldwide, ranging from diarrhea and stunting in children to impaired cognitive development from iron deficiency anemia.^1^ Globally, approximately 430 million people are infected with hookworm (*Ancylostoma duodenale* and *Necator americanus*).^2^ Among those infected with intestinal nematodes, hookworm infections are associated with the greatest years lived with disability (YLDs), with recent estimates indicating that human hookworm disease is associated with 4.1 million disability-adjusted life years.^3^ This large contribution of hookworm infections to the YLDs among those with nematode infections is due to the link between hookworm disease and anemia in children.^4–6^ Beyond its health impact, the anemia it induces is linked to a moderate economic burden ranging up to $139 billion annually.^3^

*Necator americanus* is the major hookworm species in the Americas. Transmission requires fecal contamination of soil and dermal penetration of human hosts. Conditions for larval survival, include moist and temperate environments. These soil-transmitted helminths are mainly found in areas where sanitation and hygiene are poor,^7^ most commonly in resource-limited countries. However, in the early 1930s, the southern United States had a high prevalence of hookworm infections caused by *N. americanus*. The initial surveys found 53.6% of the population to be infected with hookworm; in some areas, the prevalence rose as high as 76%.^8^ To control the disease, thousands of individuals were treated, decreasing prevalence to 39%.^9^ After these interventions, there were increases in school enrolment, attendance, and literacy, and those within the treated cohort had substantial gains in long-term incomes.^10^ However, because of posttreatment reinfection and widespread transmission, hookworm infection and disease continued to persist in the southern United States, especially in areas of extreme poverty.^11^ According to a study in the 1950s, rural Alabama still suffered from a high prevalence of hookworm infection in schoolchildren, with some counties having 60% infection.^12^ With improved sanitation and waste disposal infrastructure, in association with aggressive economic development in the southern United States, the prevalence of hookworm infection decreased. In the 1990s, surveillance studies for enteropathogens in southern Alabama show a 30% prevalence for all soil-transmitted helminths combined, including *Ascaris lumbricoides*, *N. americanus*, and *Enterobius vermicularis*.^13^ This part of the United States was identified as one at high risk for intestinal helminth infections.^14^ A more recent systematic review, however, found that few surveys for intestinal helminth infections have been conducted in recent decades, with limited information about these diseases,^15^ especially in poor rural and southern United States.

According to the Alabama Center for Rural Enterprise (ACRE), an organization that addresses poverty and economic development in one of the poorest areas of the nation,^16^ there continue to be residences without adequate sanitation systems, increasing exposure to open sewage near dwellings. The “Black-Belt” soil native to this area is composed of a firm sedimentary limestone bed overlain with a layer of dark, rich soils,^17^ which requires expensive septic systems for proper waste disposal. In Lowndes County, Alabama, where the per capita income is $18,046, and 31.4% of the population lives below the poverty line,^18^ sanitation systems are unaffordable. For rural, impoverished individuals, the main form of waste removal involves use of “straight piping,” a method involving a series of ditches or crudely constructed piping systems to guide human waste away from the residence. Most pipes never reach more than 10 meters in length, and during rainstorms or flooding, the residents report visible stool entering their homes (reported by ACRE, unpublished data).

While the prevalence of intestinal parasites has decreased in the past 85 years, conditions amenable to the parasite life-cycle and transmission persist. Because of these conditions, a more current investigation using modern molecular diagnostics is warranted to determine the burden of intestinal parasitic infection. The goal of this study seeks to address the current prevalence of helminthic and protozoan infections and determines correlation with sewage exposure in an economically challenged region of the southern United States. Given continued poverty in these areas such as Lowndes County, lack of access to contained septic systems, and potential exposure to raw sewage, intestinal helminth infection likely persists despite previous interventions. The results of this study will provide insight into disease burden within this community and a basis for further study on possible risk factors, intervention, and effects on the community.

## METHODS

### Sample population.

The study was performed in Lowndes County, an area in Alabama known to have low per capita income. According to the 2010 US Census, Lowndes County, has 5,270 houses with a 11,299 total population.^18^ ACRE reports approximately 50% of households have a failing or no sewage system (ACRE, unpublished data). Dwellings and individuals were preselected by ACRE organizers from areas with poor sanitation and used open sewage systems as a means of waste management. Enrolment goals of 100 subjects were planned, all subjects enrolled were African American. A total of 66 subjects were enrolled in the study from a total of 24 homes. There were single individuals from seven homes, four homes with two subjects, seven homes with three subjects, three homes with four subjects, one home each with five, six, and eight subjects, respectively. The research team and those assisting from ACRE visited the selected dwellings, interviewed residents using a questionnaire to obtain data regarding their dwelling and health, and collected soil from the surrounding areas and stool samples from participants. The questionnaire included information regarding sex, age, previous travel outside the United States, prior history of parasitic infections, exposure to sewage within the home, asthma, and diarrheal symptoms. Before participating in the study, each participant or guardian signed a written consent form. Participants were excluded if they have ever traveled outside the United States or were under 2 years of age, pregnant, HIV-positive, or immunocompromised. This study was approved by the Baylor College of Medicine Institutional Review Board and the Alabama Department of Public Health.

### Sample collection.

Stool samples were self-collected by the individual participants after instructions were provided verbally and in writing, and soil samples were obtained near areas of sewage collections or run-off by research team members. Participants were instructed to fill stool containers with approximately 5 grams of stool and store in provided opaque double-sealed biohazard bags in the refrigerator overnight, or if possible, the same day as the scheduled interview and collection. All samples were stored in dry ice coolers after collection and transported to the Laboratory of Clinical Parasitology and Diagnostics, Baylor College of Medicine within 5 days of collection, and stored in −20°C freezers.

### Molecular methods.

DNA was extracted from the frozen stool samples the following week after collection using MP FastPrep^®^ spin kits for soil (MP Biomedicals, Santa Ana, CA) after a modified method was developed by the primary investigator, as previously described, for *Trichuris trichiura* DNA.^19^ Parasite DNA was detected using a multiparallel quantitative real-time PCR (qPCR) protocol.^19^ Species-specific primers and 6-carboxyfluorescein-labeled Minor groove binder probes (Applied Biosystems, Foster City, CA) were selected for each of the eight parasites tested, including helminths (*Ascaris lumbricoides*, *Trichuris trichiura*, *Strongyloides stercoralis*, *Ancylostoma duodenale*, and *N.americanus*), and protozoa (*Giardia lamblia*, *Cryptosporidium* species, and *Entamoeba histolytica*)^19^ (Supplemental Table 1). Samples were analyzed on an ABI ViiA 7 Real-Time PCR System (Applied Biosystems) using default parameters for fast chemistry and 40 cycles.^19^ DNA concentrations were calculated using parasite plasmid standard curves.^19^ All controls (positive, negative, and a pBR322 plasmid internal control) were performed in triplicate with subject samples in duplicate. Samples were repeated for discordant results. The threshold for positivity was set at 38 cycle thresholds (Ct), which was the limit of detection for our dynamic range of positive standard curves.^19^ Fifty grams of top soil was collected at sites close to the opening of “straight pipes,” and the same protocol used for stool was implemented for extracting DNA from soil samples.

### Additional analysis.

A subsequent serological evaluation using an enzyme-linked immunosorbent assay (ELISA) protocol for the detection of *Toxocara* species was performed on 11 patients who were found to be positive for *N. americanus* and had serum available for study. Because of logistical restraints, blood from only those positive for any parasite was requested, and only 11 subjects consented. A commercially available *Toxocara* ELISA was used per manufacturer protocol (Abnova, Taiwan) with positive absorbance readings accepted as greater than or equal to 0.3 optical density units. For the detection of *Strongyloides stercoralis*, a previously described NIE-ELISA protocol^20^ was implemented using serum from the same 11 participants who were tested for *Toxocara* species. Unfortunately, subject’s stool positive for *Strongyloides* by qPCR declined further serum work up. Any positive laboratory result (qPCR, microscopy, ELISA) was communicated to the subject and their primary care provider, who decided what clinical course to take.

Gel electrophoresis with 4% agarose gel was also used in this study as a confirmatory tool for positive results. Sample standards were used, as well as positive and negative controls. Bands were visualized by ultraviolet fluorescent detection. Those testing positive for *N. americanus* infection had their stool sent to the Centers for Disease Control (CDC) for ova and parasites, and concentration microscopic evaluation.

### Data analysis.

All statistical analyses were performed using Prism software version 5.0d (GraphPad, La Jolla, CA). The level of significance was set at *P* < 0.05 for all analyses, and all statistical tests were two-sided. Descriptive statistics were computed to describe data for sample characteristics. Frequency distribution and measures of central tendency and dispersion were expressed by geometric means, medians, standard deviations, and proportions. Fisher’s exact test was used to compare categorical variables and proportions. All qPCR positive samples were reported as parasite DNA concentrations measured in femtograms per microliter (fg/μL). Data were generated by detecting which parasites were present in each person. Linear regression was used to calculate fg/μL to hookworm eggs per gram (epg) of stool by microscopy in a previous study, which provides a good representation of parasitic infections within a population.^21^

## RESULTS

**Figure 1.**
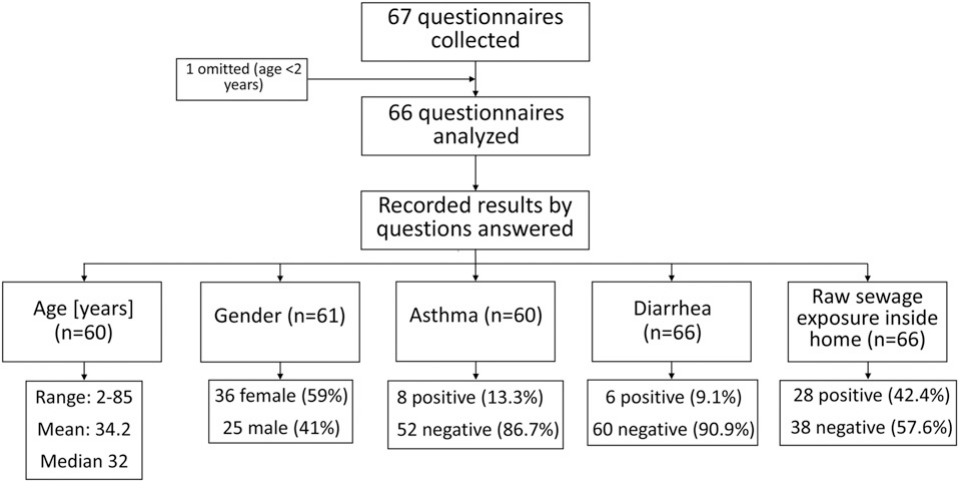
Flow diagram of questionnaires collected, questions answered, and results of the data available.

The questionnaires were collected for 67 individuals, but given exclusion criteria, only 66 were included in this study. Not all questions were answered, therefore, the questionnaire results were evaluated separately in a subgroup analysis (Figure 1). Age ranged from 2 to 85 years, with a mean of 34.2 years of age. The participants included 36 females and 25 males. No one reported a previous diagnosis of a parasitic infection. Only six individuals reported having diarrhea in the past 7 days, and eight reported having asthma in the past 30 days. Twenty-eight individuals reported having exposure to raw sewage inside their domicile (42.4% of those answering the questionnaire within this study population).

**Figure 2.**
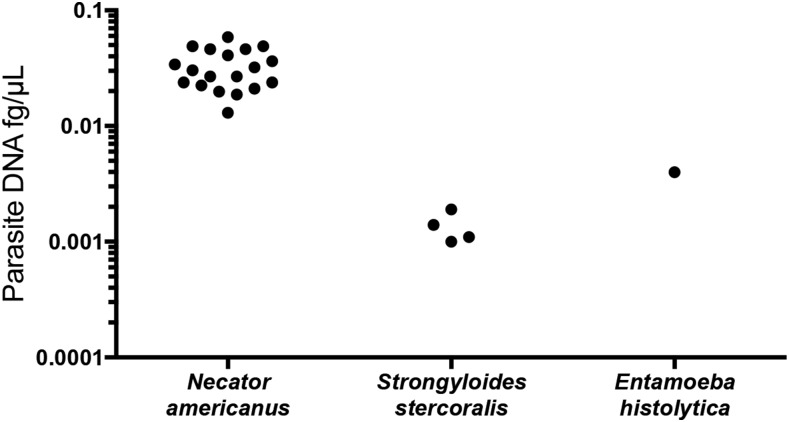
Parasite DNA fg/µL in positive stool samples from rural Alabama.

Stool samples were collected for 55 individuals. Of these, 19 (34.5%) were positive for *N. americanus*, four (7.3%) for *Strongyloides stercoralis*, and one (1.8%) for *Entamoeba histolytica* (Figure 2). Polyparasitism was identified in two stool samples, both of which were positive for *N. americanus* and *Strongyloides stercoralis*. All 55 samples were negative for *Giardia lamblia*, *Cryptosporidium* species, *Ascaris lumbricoides*, and *Trichirus trichiura*. Stool samples positive for *N. americanus* contained low levels of parasite DNA (range of 0.013–0.059 fg/μL), and the estimated epg, based on calculations from a previous study,^21^ ranged from 0.474 to 2.14 epg of stool. Microscopy performed on qPCR positive stool samples were negative for ova or parasites. The 19 *N. americanus* positive samples detected by qPCR were run on 4% agarose gel electrophoresis with sample standards, and all 19 samples had bands corresponding to the appropriate size using a DNA ladder (New England Biolabs, Ipswich, MA) (Supplemental Figure 1). None of the hookworm samples were positive for *Ancylostoma duodenale*.

Eleven of the 23 participants with stool samples positive for any parasite consented to further serology assays. Of these 11 participants, one (5.2%) tested positive for *Toxocara* species. None tested positive for *Strongyloides stercoralis* IgG antibodies based on ELISA. Of the 34 soil samples collected, one (2.9%) tested positive for the protozoa, *Cryptosporidium* species.

Further subanalysis was performed on 26 individuals with fully completed questionnaires and qPCR stool testing. Those meeting these criteria included 10 males and 16 females. Stool samples tested negative for 15 patients, *N. americanus* positive for 11 patients, and *N. americanus* and *Strongyloides stercoralis* coinfection positive for two patients. Females comprised 61.5% of this subgroup analysis. There was no statistically significant difference between males and females with or without *N. americanus* infection (*P* = 0.68) (Table 1). Raw sewage was reported inside the home for 73.1% of individuals included within this subanalysis, but there was no statistically significant difference between those testing positive and negative for *Necator americanus* (*P* = 0.61). Three individuals reported a diagnosis of asthma, and one reported an episode of diarrhea within the past 7 days, but were not statistically different between the uninfected and those infected with *N. americanus* (*P* ≥ 0.99) (Table 1).

**Table 1:** Questionnaire subanalysis for patients with fully completed questionnaires and qPCR stool testing

Characteristic	Parasite infection			*P* value†
	Uninfected [*N* = 15 (%)*]	*Necator* only infection [*N* = 9 (%)*]	Any infection [*N* = 11 (%)*]	
Sex (*N* = 26)				
Male 10 (38.5%)	5 (33.3%)	4 (44.4%)	5 (45.5%)	0.679
Female 16 (61.5%)	10 (66.7%)	5 (55.6%)	6 (54.5%)	–
Raw sewage in home	11 (73.3%)	8 (88.9%)	8 (72.7%)	0.615
Asthma or other lung disease	3 (20.0%)	2 (22.2%)	2 (18.2%)	> 0.999
Diarrhea in past week	1 (6.7%)	1 (11.1%)	1 (9.1%)	> 0.999

* Percentage of individuals within each infection group.

† *P* values derived from Fisher’s exact test for association between uninfected and *Necator* infection. All *P* values were above 0.05 comparing uninfected to any infection groups.

## DISCUSSION

Using field-tested molecular assays, 34.5% of individuals living in a high-risk environment within a region of the southern United States were found to have stool samples testing positive for *N. americanus* (hookworm). Even though the parasite burden was low, with up to 0.059 fg/μL of parasite DNA and an estimated egg burden up to 2.1 epg of stool, there is evidence of endemic and autochthonous infection within this population. In addition, 73% of Lowndes County residents who were tested and completed the questionnaire reported exposure to raw sewage inside their home, and even though not statistically significant (likely due to small sample size), 72.7% with this exposure tested positive for parasitic infection. By using qPCR, gastrointestinal parasites known to be endemic to developing countries have now been identified in a resource-limited county in Alabama, among people who have never traveled abroad, likely from autochthonous infection.

Despite decreased hookworm prevalence reported by previous soil helminth eradication programs, infection is still present within this population. During the last century, repeat surveys were performed to track the improvement of helminth infections among local populations, but these studies used microscopy as the diagnostic modality, which is limited by time requirements, manpower, and need for skilled microscopists. Even among those with ample training and experience within the field of microscopy, sensitivity for ova and parasite detection is 50–85%.^22^ In addition, detection of ova and parasites is severely limited by the burden of disease—those with less parasite burden are less likely to test positive via microscopy. Given these limitations, this study was unable to provide a correlation between the detection of parasite DNA and the microscopy analysis of qPCR positive stool samples. This was most likely due to the low *N. americanus* egg burden (calculated 1–2 epg of stool), which is below the microscopy limits of detection (12 epg).^21,23,24^ These limitations further justify the utility of qPCR as a method for detection of intestinal parasite infections within these resource-limited communities, where the burden of disease might be too low to accurately detect via microscopy. A new method for parasite detection could provide a more sensitive diagnostic approach for those within these communities with a continued risk of low hookworm burden and other parasitic infections. These constraints explain why qPCR is a desirable method to use with the potential of multiplexing samples for varied infections.^23^ Moreover, qPCR requires less skilled operators, and has a significantly higher sensitivity for detection of parasite DNA, especially those with a low burden of disease, detecting levels as low as 100 attograms [1 × 10^−18^] of DNA.^19,21^ The limit of detection for qPCR and its specificity effectively rule out *A. duodenale* hookworm DNA in the samples, together with a historical record showing an absence of *A. duodenale* infections in the southern United States. Together with the positive gel bands (Supplemental Figure 1), strengthens the likelihood that the positive DNA signals for *N. americanus* hookworm in this study do not reflect false positives and that transmission continues in the modern era.

As shown in the 1950s Alabama study, hookworm infection rates were as high as 60% in some of the more poverty-stricken communities. In association with overall economic development in the years during and after Franklin Delano Roosevelt’s New Deal legislation, together with improvements in sanitation, urbanization, and industrialization, these pockets of infection were thought to have resolved.^9^ However, given continued poor sanitation and advancement of detection methods (improved sensitivity with PCR compared with microscopy), low burdens of infection have now been discovered in the United States among populations with autochthonous transmission. Further testing is necessary as hookworm continues to be a problem in areas with poor sanitation, allowing recurrent infections due to repeat exposure. Other areas in the United States have also been found to harbor significant pockets of parasitic infections thought only endemic to developing countries. *Strongyloides stercoralis* has also been found in some Appalachian mountain communities,^25^ as well as in Kentucky^26^ and rural Tennessee.^27^ Interestingly, we detected a 5.2% *Toxocara* IgG serological prevalence, which coincides with predicted national prevalence for this parasite among African Americans.^28^ While the soil sampling did not reveal the presence of helminths, this was likely because of the limitations of random sampling; however a single *Cryptsporidium* species sample was detected and reinforces the perception that human or animal fecal contamination occurs near these houses. Another limitation of soil was that no concentration steps were performed, only 50 mg of soil per 50 gm soil sample was processed for DNA extraction. This small amount of soil and low sample numbers likely contributed to low-parasite positive samples. The discovery of these parasitic diseases within the United States begins to shift the idea behind global health. One concept is blue marble health,^29^ which reveals that many of the world’s neglected tropical diseases are paradoxically found in some of the wealthiest countries, especially in these regions of extreme poverty. With the introduction of more advanced diagnostic techniques, emergence of rare, endemic infections may eventually become less defined by geographic location, but more by economic status.

Unfortunately, because of the mistrust stemming from the illegalities of the self-constructed “straight pipe” waste disposal systems, as well as toward the medical community, the number of individuals included in this study was much smaller than expected. By working with the ACRE organization, which has fostered trust and worked with several members within the community, the research team was able to include more participants, but some of the data obtained from the questionnaires were incomplete because of different individuals performing the interviews. This should be considered when interpreting the results of this study. Performing subgroup analyses with the information provided some baseline data that could be assessed and compared among those being tested for infection. These incomplete forms were still included in data analyses given the difficulty of obtaining this information by other means and to provide further insight into the local population.

This preliminary study demonstrates that gastrointestinal parasites are present in > 30% of this at-risk population in Lowndes County, Alabama. Further testing in this community is vital to better comprehend the parasitic burden in this population, and additional studies should be initiated to further understand the implications and effects on health and the community. Parasitic testing needs to be expanded to include more households to determine the prevalence and quantitative parasitic burden. Hemoglobin monitoring should be performed among those testing positive for infection, against a negative control group, to determine if there is an impact on health with low parasitic burdens or a correlation between parasitic burden and hemoglobin levels. Hookworm (*N. americanus*) has also been shown to impair immune recovery (CD4^+^ cells) in HIV-infected individuals by, an average of, 102 cells/μL fewer CD4^+^ cells in those infected with *Necator*.^30^ Interestingly, the participants of that study also had low *Necator* burden of infection (0.025 fg/μL, 0.72 epg) and similar to the Alabama findings.^30^ According to 2014 data, the rates of HIV infection in Lowndes county is 758 per 100,000 African Americans and is an area with some of the highest rates of HIV infections in the United States.^31^ The combination of HIV and subclinical hookworm infections in Alabama could have an impact on immune recovery. Also, previous studies found a correlation between treatment and substantial gains in long-term income, as well as improvement of school enrolment, attendance, and literacy after hookworm eradication programs.^10^ By monitoring for changes in median income, standard of living, and economic status within this population after treatment, correlations of these socioeconomic endpoints to infection, even among those with a low burden of disease, could be determined. Further understanding of the disease process and parasitic burden is vital for future public health initiatives, decreasing hookworm infection, treatment, and improvement of health outcomes, with the overall goal of eradicating this neglected tropical disease in the United States and worldwide.

## Supplementary Material

Supplemental Figure and Table.

## References

[1] Checkley W Buckley G Gilman RH Assis AM Guerrant RL Morris SS Melbak K Valentiner-Branth P Lanata CF Black RE, 2008 Multi-country analysis of the effects of diarrhoea on childhood stunting. Int J Epidemiol 37: 816–830. 18567626 10.1093/ije/dyn099 PMC2734063

[2] GBD 2015 Disease and Injury Incidence and Prevalence Collaborators, 2016 Global, regional, and national incidence, prevalence, and years lived with disability for 310 diseases and injuries, 1990–2015: a systematic analysis for the Global Burden of Disease Study 2015. Lancet 388: 1545–1602. 27733282 10.1016/S0140-6736(16)31678-6 PMC5055577

[3] Bartsch SM Hotez PJ Asti L Zapf KM Bottazzi ME Diemert DJ Lee BY, 2016 The global economic and health burden of human hookworm infection. PLoS Negl Trop Dis 10: e0004922. 27607360 10.1371/journal.pntd.0004922 PMC5015833

[4] Brooker S Hotez PJ Bundy DAP, 2008 Hookworm-related anaemia among pregnant women: a systematic review. PLoS Negl Trop Dis 2: e291. 18820740 10.1371/journal.pntd.0000291 PMC2553481

[5] Smith JL Brooker S, 2010 Impact of hookworm infection and deworming on anaemia in non-pregnant populations: a systematic review. Trop Med Int Health 15: 776–795. 20500563 10.1111/j.1365-3156.2010.02542.x PMC2916221

[6] Hotez PJ Brooker S Bethony JM Bottazzi ME Loukas A Xiao S, 2004 Hookworm infection. N Engl J Med 351: 799. 15317893 10.1056/NEJMra032492

[7] CDC, Global Health - Division of Parasitic Diseases, 2013 *Parasites.* Available at: https://www.cdc.gov/parasites/sth/. Accessed December 15, 2016.

[8] Smith WHY McAlpine JG Gill DG, 1937 Intestinal parasite survey in Alabama. A comparative study of two hookworm anthelmintics. Am J Public Health 27: 471–475. 10.2105/ajph.27.5.471 PMC1563166 18014623

[9] Humphreys M, 2009 How four once common diseases were eliminated from the American south. Health Aff 28: 1734–1744. 10.1377/hlthaff.28.6.1734 19887414

[10] Bleakley H, 2007 Disease and development: evidence from hookworm eradication in the American south. Q J Econ 122: 73–117. 24146438 10.1162/qjec.121.1.73 PMC3800113

[11] Hotez P, 2007 Hookworm and poverty. Ann N Y Acad Sci 1136: 38–44. 17954674 10.1196/annals.1425.000

[12] Hosty TS Wells DM Freear MA Whitfield NK, 1954 Hookworm in Alabama. J Med Assoc State Ala 23: 179–182. 13118326

[13] Badham AL, 1993 *Wilcox County Alabama: Needs Assessment, Doctoral Dissertation.* Birmingham, AL: University of Alabama at Birmingham. Available at: https://assets.documentcloud.org/documents/2091580/pdf1-hookworm1993.pdf. Accessed December 15, 2016.

[14] Hotez PJ, 2008 Neglected infections of poverty in the United States of America. PLoS Negl Trop Dis 2: e256. 18575621 10.1371/journal.pntd.0000256 PMC2430531

[15] Starr MC Montgomery SP, 2011 Soil-transmitted helminthiasis in the United States: a systematic review-1940–2010. Am J Trop Med Hyg 85: 680–684. 21976572 10.4269/ajtmh.2011.11-0214 PMC3183777

[16] ACRE, Alabama Center for Rural Enterprise CDC Inc., 2017 *ACRE CDC, Inc. Overview* Available at: http://acrecdc.com/. Accessed May 15, 2017.

[17] Winemiller TL, 2009 *Black Belt Region in Alabama. Encyclopedia of Alabama* Auburn University at Montgomery. Available at: http://www.encyclopediaofalabama.org/article/h-2458. Accessed December 15, 2016.

[18] United States Census Bureau, 2010–2014 *Lowndes County Alabama: Income and Poverty* Available at: http://www.census.gov/quickfacts/table/INC110214/01085. Accessed December 15, 2016.

[19] Mejia R Vicuña Y Broncano N Sandoval C Vaca M Chico M Cooper PJ Nutman TB, 2013 A novel, multi-parallel, real-time polymerase chain reaction approach for eight gastrointestinal parasites provides improved diagnostic capabilities to resource-limited at-risk populations. Am J Trop Med Hyg 88: 1041–1047. 23509117 10.4269/ajtmh.12-0726 PMC3752800

[20] Nabha L Krishnan S Ramanathan R Mejia R Roby G Sheikh V McAuliffe I Nutman T Sereti I, 2012 Prevalence of *Strongyloides stercoralis* in an urban USAIDS cohort. Pathog Glob Health 106: 238–244. 23265425 10.1179/2047773212Y.0000000031 PMC4001591

[21] Cimino RO Jeun R Juarez M Cajal PS Vargas P Echazú A Bryan PE Nasser J Krolewiecki A Mejia R, 2015 Identification of human intestinal parasites affecting an asymptomatic peri-urban Argentinian population using multi-parallel quantitative real-time polymerase chain reaction. Parasit Vectors 8: 380. 26183074 10.1186/s13071-015-0994-z PMC4504406

[22] Mosli M Gregor J Chande N Lannigan R, 2012 Nonutility of routine testing of stool for ova and parasites in a tertiary care Canadian centre. Can J Microbiol 58: 653–659. 22540249 10.1139/w2012-039

[23] Basuni M Mohamed Z Ahmad M Zakaria NZ Noordin R, 2012 Detection of selected intestinal helminths and protozoa at Hospital Universiti Sains Malaysia using multiplex real-time PCR. Trop Biomed 29: 434–442. 23018507

[24] Verweij JJ Brienen EA Ziem J Yelifari L Polderman AM Van Lieshout L, 2007 Simultaneous detection and quantification of *Ancylostoma duodenale, Necator americanus*, and *Oesophagostomum bifurcum* in fecal samples using multiplex real-time PCR. Am J Trop Med Hyg 77: 685–690. 17978072

[25] Segarra-Newnham M, 2007 Manifestations, diagnosis, and treatment of *Strongyloides stercoralis* infection. Ann Pharmacother 41: 1992–2001. 17940124 10.1345/aph.1K302

[26] Fulmer HS Huempfner HR, 1965 Intestinal helminths in eastern Kentucky: a survey in three rural counties. Am J Trop Med Hyg 14: 269–275. 14270453 10.4269/ajtmh.1965.14.269

[27] Berk SL Verghese A Alvarez S Hall K Smith B, 1987 Clinical and epidemiologic features of strongyloidiasis. A prospective study in rural Tennessee. Arch Intern Med 147: 1257–1261. 3606282

[28] Congdon P Lloyd P, 2011 *Toxocara* infection in the United States: the relevance of poverty, geography and demography as risk factors, and implications for estimating county prevalence. Inter J Pubc Health 56: 15–24. 10.1007/s00038-010-0143-6 20422250

[29] Hotez PJ, 2013 NTDs V.2.0: “Blue Marble Health”-neglected tropical disease control and elimination in a shifting health policy landscape. PLoS Negl Trop Dis 7: e2570. 24278496 10.1371/journal.pntd.0002570 PMC3836998

[30] Morawski BM Yunus M Kerukadho E Turyasingura G Barbra L Ojok AM DiNardo AR Sowinski S Boulware DR Mejia R, 2017 Hookworm infection is associated with decreased CD4^+^ T cell counts in HIV-infected adult Ugandans. PLoS Negl Trop Dis 11: e0005634. 28542260 10.1371/journal.pntd.0005634 PMC5462474

[31] Sullivan P, 2017 *AIDSVu Interactive Online HIV Prevalence Map Based on U.S. Centers for Disease Control and Prevention’s National HIV Surveillance Database.* Available at: https://aidsvu.org. Accessed June 30, 2017.

