# Erratum

**DOI:** 10.4269/ajtmh.17-0396err

**Published:** 2018-03

In *Am J Trop Med Hyg 97*(5): 1623–1628 by McKenna et al (https://doi.org/10.4269/ajtmh.17-0396) a line is missing from the financial support section. It should say “McKenna ML received support from the National Institutes of Health (grant #T32 AI 055413).”

