# Supplementary material for: Human Intestinal Parasite Burden and Poor Sanitation in Rural Alabama
Source: Am J Trop Med Hyg. 2017 Sep 5;97(5):1623–8. doi: 10.4269/ajtmh.17-0396 (PMC5817782; doi:10.4269/ajtmh.17-0396)
Supplement: Supplementary file 1 [file tpmd170396.SD1.pdf]

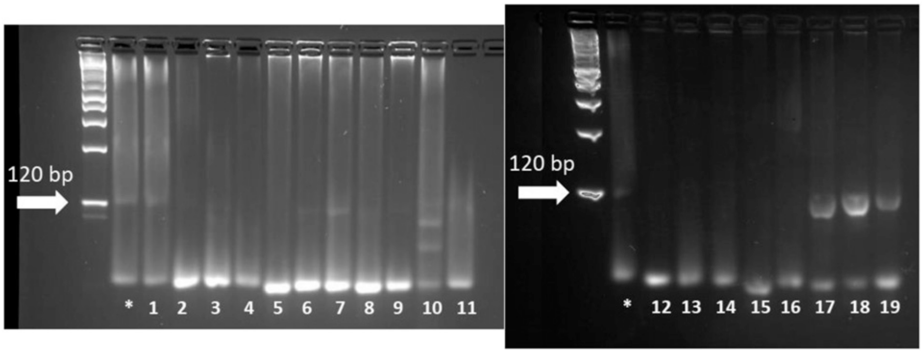

Supplemental Figure 1. DNA gels showing size matching of 19 samples to 120 base pairs from ladder and *Necator* plasmid standards. All samples have bands, but some are very light and difficult to visualize on these images.

Supplemental Table 1
Primer, probes, and target regions for all eight parasites. Sequence information for the eight parasites

Parasite	Forward primer sequence Reverse primer sequence probe sequence (FAM)	Target region	Gene ascension number
Ascaris lumbricoides	5′-TGCACATAAGTACTATTTGCGCGTAT-3′<br>5′-CCGCCGACTGCTATTACATCA-3′<br>5′-GAGCCACATAGTAAATT-3′	ITS-1	AB571301.1
Cryptosporidium Species	5′-AACTTCACGTGTGTTTGCCAAT-3′<br>5′-CCAATCACAGAATCATCAGAATCG-3′<br>5′-CATATGAAGTTATAGGGATACCAG-3′	DNAJ like protein	XM_625506.1
Ancylostoma duodenale	5′-GAATGACAGCAAACTCGTTGTTG-3′<br>5′-ATACTAGCCACTGCCGAAACGT-3′<br>5′-ATCGTTTACCGACTTTAG-3′	ITS-2	EU344797.1
Necator americanus	5′-CTGTTTGTCGAACGGTACTTGC-3′<br>5′-ATAACAGCGTGCACATGTTGC-3′<br>5′-CTGTACTACGCATTGTATAC-3′	ITS-2	AJ001599.1
Strongyloides stercoralis	5′-GAATTCCAAGTAAACGTAAGTCATTAGC-3′<br>5′-TGCCTCTGGATATTGCTCAGTTC-3′<br>5′-ACACACCGGCCGTCGCTGC-3′	18S rRNA	AF279916.2
Giardia lamblia	5′-CATGCATGCCCGCTCA-3′<br>5′-AGCGGTGTCCGGCTAGC-3′<br>5′-AGGACAACGGTTGCAC-3′	16S rRNA	AJ293299.1
Entamoeba histolytica	5′-GTTTGTATTAGTACAAAATGGCCAATTC-3′<br>5′-TCGTGGCATCCTAACTCACTTAGA-3′<br>5′-CAATGAATTGAGAAATGACA-3′	18S rRNA	X75434.1
